# Alpha thalassemia genotypes in Kuwait

**DOI:** 10.1186/s12881-020-01105-y

**Published:** 2020-08-24

**Authors:** Adekunle Adekile, Jalaja Sukumaran, Diana Thomas, Thomas D’Souza, Mohammad Haider

**Affiliations:** Department of Pediatrics, Faculty of Medicine, Kuwait University, PO Box 24923, 13110 Safat, Kuwait

**Keywords:** Alpha thalassemia, Hemoglobin H disease, Kuwait

## Abstract

**Background:**

The frequency of the alpha thalassemia trait is approximately 40% in the Kuwaiti population, but there has been no comprehensive study of the prevalent alleles. This is a report of patients who were referred for molecular diagnosis over a 20-year period.

**Methods:**

This is a retrospective study of the α-globin genotypes obtained in the Hemoglobin Research Laboratory of the Department of Pediatrics, Kuwait University from 1994 to 2015. Genotyping was performed by a combination of PCR, allele-specific oligonucleotide hybridization and reverse dot blot hybridization (Vienna Lab Strip Assay).

**Results:**

Four hundred samples were characterized and analyzed from individuals aged < 1 month to 80 years, with a median of 6 years from 283 unrelated families. Most (90.8%) were Kuwaiti nationals. The commonest genotype was homozygosity for the polyadenylation-1 mutation (α^PA-1^α/α ^PA-1^α) in 33.3% of the samples, followed by heterozygosity (αα/α ^PA-1^α) for the same mutation in 32.3%. PA-1 was therefore the most frequent allele (0.59). The frequency of the α^0^ (−-^MED^) allele was 0.017. Rare alleles that were found in very low frequencies included α^0^ (−-^FIL^) in a Filipino child, Hb Constant Spring, Hb Adana, and Hb Icaria.

**Conclusion:**

There is a wide variety of alpha thalassemia alleles among Kuwaitis, but nondeletional PA-1 is by far the most common cause of the moderate to severe HbH (β4 tetramer) disease phenotype. The α^0^ (−^MED^) allele is also encountered, which has implications for premarital counseling, especially for the possibility of having babies with alpha thalassemia major (Barts hydrops fetalis).

## Background

Alpha thalassemia is one of the most widespread genetic diseases worldwide, with frequencies of the carrier state reaching up to 80–90% in some areas [1]. The incidence is approximately 25–30% in tropical Africa [2], while in the Arabian Peninsula, the frequency varies from a low of close to zero in the desert areas to as high as 60% in the agricultural zones of Eastern Saudi Arabia [3, 4].

The normal individual has a complement of four α-globin genes (αα/αα), any one of which can be absent, producing varying degrees of alpha thalassemia [1]. In α^0^ alleles, both genes on a chromosome are deleted (--), while in α^+^, a variable portion of the α2 and/or α1 gene is deleted, resulting in a reduction in chain synthesis. The size of the deletion in α^0^ alleles is variable, and each is named for the part of the world where it is prevalent. Thus --^MED^,(--)^FIL^, and --^SEA^ deletions are found in the Mediterranean, Philippines and South East Asia, respectively [5]. The α^+^ deletions are designated by their sizes, and the most common is the –α^-3.7 kb^ allele. One- or 2-gene deletions (−α/αα, −α/−α, −−/αα) produce the α-thal trait, which is usually associated with mild microcytic and hypochromic anemia. The loss of 3 genes (−−/−α) produces classical hemoglobin H (HbH, β4 tetramer) disease, which is characterized by moderate to severe anemia. The loss of all 4 genes (−−/−−) causes Hb Barts (γ4) hydrops fetalis, which is incompatible with life in the absence of the early onset of chronic transfusion or stem cell transplantation [6].

Apart from deletions, there are a few point mutations that affect the transcriptional efficiency of the α-globin genes and are designated α^T^α or αα^T^ depending on whether the α2 or α1 gene is affected [7]. These nondeletional α-thalassemia alleles commonly affect the promoter, initiation codon (ATG), splicing signals (GT/AG), termination codon (TAA), and polyadenylation signal (AATAAA) of the affected gene. The most common of these are α^IVSI(−5nt)^ α (in the Mediterranean deletion), polyadenylation site mutations α_2_^AATAAG^, α_2_^AATGAA^ and α_2_^AATA--^ (in the Mediterranean and Middle East deletions) [7–11], and termination codon mutations leading to elongated Hb variants, such as Hb Constant Spring (HbCS), Hb Icaria, Hb Koya Dora, Hb Seal Rock and Hb Pakse [12–14]. Heterozygotes for these alleles have a thalassemia minor phenotype, but homozygotes and compound heterozygotes often have HbH disease of varying severity and blood transfusion requirements.

The α-thalassemia frequency is approximately 30–40% in Kuwait, and HbH disease is mostly linked to the polyadenylation (polyA, α^PA-1^) mutation [15, 16]. However, there has been no comprehensive study of the spectrum of alpha thalassemia alleles in the country. The present study was carried out to document the frequencies of moderate to severe alpha thalassemia phenotypes among patients referred for molecular diagnosis. In particular, it aimed to investigate the presence of the α^0^ allele in the population and to determine the possibility of having a newborn with alpha thalassemia major (Hb Bart’s hydrops fetalis) [1, 17]. This will enable more purposeful counseling of patients and empower physicians in the identification of patients who require further investigations. To this end, we analyzed all cases of suspected α-thalassemia referred to the hemoglobin research laboratory at Kuwait University over a 20-year period.

## Methods

The hemoglobin research laboratory in the Department of Pediatrics, Kuwait University received samples from the hematology clinics in Mubarak Al-Kabeer Hospital and some of the other government hospitals in Kuwait. The study was approved by the Human Research Ethics Committee of the Faculty of Medicine, Kuwait University and the Ministry of Health, Kuwait. Patients were suspected to have some form of α-thalassemia if they had persistent microcytic and hypochromic anemia in the absence of iron deficiency and normal hemoglobin electrophoresis. Some patients had H inclusions on cresyl blue staining of the peripheral erythrocytes or an H band on high-performance liquid chromatography (HPLC). Quite often, but not always, there was a record of the clinical presentation and the complete blood count (CBC). This study is a retrospective study of all such samples analyzed from 1994 to 2005.

Blood was obtained by venipuncture into EDTA tubes, and a complete blood count was performed using an ABX Pentra 120 cell counter (ABX France, Montpellier). Hemoglobin quantitation was determined using cation-exchange high-performance liquid chromatography (HPLC) (Shimadzu LC-20AT, Shimadzu Corporation, Kyoto, Japan). DNA was isolated from peripheral leucocytes using phenol extraction. All the samples were screened for the α-thal-2 (− 3.7 kb) deletion [18] and the α2-globin gene polyadenylation site *(AATAAA→AATAAG)* mutation using a PCR method and allele-specific oligonucleotide hybridization, respectively, as previously reported [18, 19]. All other samples that were not resolved with these methods were subjected to reverse dot blot hybridization using the Vienna Lab StripAssay technique (Vienna Lab Diagnostics, Vienna, Austria).

The ethnic origins of the patients were documented by their nationalities. The different α-globin genotypes were identified, and the allele frequencies were calculated. The mean Hb level and RBC indices were compared among patients with different genotypes. Clinical data were collected from the HbH disease patients, especially whether they were being followed in a hematology clinic and if they were on any medications or how often they were transfused with blood.

The disease severity was classified as silent carrier, α-thalassemia trait or HbH disease depending on the number, type and location of mutations detected, in addition to the clinical phenotype and presence of HbH inclusions or H bands on HPLC [1, 17, 20].

Data are presented as the means ± SD or as percentages, as appropriate. Statistical differences between mean values among the major groups were tested using Student’s t test. All descriptive analyses were performed with the International Business Machines Corporation Statistical Package for Social Sciences (IBM SPSS Statistics, New York, USA), version 25 software. The criterion for statistical significance was *p* < 0.05.

## Results

Four hundred blood samples were received with the diagnosis of suspected moderate to severe alpha thalassemia or HbH disease over the period of the study from 1994 to 2015. There were 183 females and 217 males, with ages ranging from 1 to 80 years and a median of 6 years. Table 1 shows the nationalities of the patients in whom these were documented; most (90.8%) were Kuwaiti. The patients were from 283 unrelated families.

**Table 1:** Nationalities of the Patients in the study

Nationality	n	%
Kuwaiti	317	90.8
Iraqi	10	2.9
Egyptian	6	1.7
Lebanese	4	1.1
Non Kuwaiti Bedouin	3	0.9
Syrian	3	0.9
Others	6	1.7
Total	349*	100.0

*These are all the patients in whom nationality was documented

Table 2 shows the distribution of α-globin genotypes in the study. The most common (33.3%) was homozygosity for α^PA-1^ (c.*94A > G) nondeletional α-thal allele, which gives an HbH disease phenotype, followed by heterozygosity for the same allele (32.3%). The compound heterozygosity of –α^-3.7^/α^PA-1^ was the next most common at 20.5%. None of the other frequencies reached 10%.

**Table 2:** Distribution of Alpha Globin Genotypes of the Patients in the Study

Genotype	Number	%
α^PA-1^α/α^PA-1^α	133	33.3
α^PA-1^α/αα	129	32.3
−α^3.7^/α^PA-1^α	81	20.5
−α^3.7^/−α^3.7^	20	5.0
--^MED^/α^CD19^α	6	1.5
α^CD59^α/ αα	4	1.0
-α^3.7^/ αα	3	0.8
--^MED^/αα	3	0.8
α^5nt^α/α^5nt^α	3	0.8
−α^3.7^/α^5nt^α	2	0.5
−α^3.7^/−−^MED^	2	0.5
αα/α^CD19^α	2	0.5
α^5nt^α/α^PA-1^α	1	0.3
--^MED^/α^5nt^α	1	0.3
--^MED^/α^PA-1^α	1	0.3
α^CS^α/α^CD142^α	1	0.3
α^PA-2^α/αα	1	0.3
α^5nt^α/αα	1	0.3
-α^4.2^/−α^4.2^	1	0.3
α^CD19^α/α^CD19^α	1	0.3
α^PA1^α/α^PA2^α	1	0.3
α^CD59^α/αα	1	0.3
α^CD59^α/α^PA-1^α	1	0.3
α^CD59^α/α^5nt^α	1	0.3
--^SEA^/−α^3.7^	1	0.3
Total	400	100

Three (0.8%) patients were classified as silent carriers, 154 (38.5%) as α-thalassemia trait, and 243 (60.8%) had HbH disease. Table 3 shows the Hb and RBC indices in the 3 groups of patients. When values were compared between the patients with α-thal trait and those with HbH disease, all indices were significantly lower in the latter (*p* < 0.05), although the mean Hb was not lower than 10 g/dl in all groups, showing that the α-thalassemia phenotypes were relatively mild in this population. None of the patients were on chronic transfusion therapy, but many had received sporadic transfusions. The patients with the most severe phenotypes in the study were 2 siblings with the --^MED^/α^cd19^α genotype. They both had poor growth parameters and baseline Hb of < 6 g/dl and are now transfused every 6 to 8 weeks. Interestingly, they have 2 other affected siblings (included in Tables 2 and 4) who do not require transfusions.

**Table 3:** Red Blood Cell Indices in Patients

Group	Hb g/dl	MCV fl	MCH pg
Silent Carrier	10.4 ± 0.8	60.7 ± 9.5	19.8 ± 2.5
α-Thalassemia Trait	11.3 ± 2.0	64.4 ± 11.0	21.1 ± 3.5
HbH	10.0 ± 1.6	59.2 ± 8.7	18.3 ± 3.1
Total	10.5 ± 1.9	61.2 ± 10.0	19.4 ± 3.5

**Table 4:** Frequency of the Common Alpha Thalassemia Alleles in the Study

	All population		Kuwaiti	
Allele	*N* = 698	Frequency	*N* = 634	Frequency
AATAAA➔AATAAG (PA-1)	412	0.590	386	0.608
-α-3.7 single gene deletion	107	0.153	91	0.143
--MED double gene deletion	12	0.017	12	0.019
α2 cd19 (−G)	10	0.0143	9	0.0141
α2 IVS-1 (−5 nt)	8	0.011	5	0.008
α2 cd59 (G➔A)	4	0.006	4	0.006
-α-4.2 single gene deletion	2	0.003	2	0.003
AATAAA➔AATGAA (PA-2)	2	0.003	2	0.003
α2 cd 142 (T➔C) Hb Constant Spring	2	0.003	2	0.003

Table 4 shows the frequencies of the common individual alleles in the whole population and among the Kuwaiti individuals in the study. The PA-1 allele was the most frequent, at ~ 0.60 in both groups, followed by the -α^-3.7 kb^ deletion at 0.153. Interestingly, α^0^–^MED^ (0.019), −α^-4.2^, α2^cd 142 (T➔C)^ (Hb Constant Spring, c.427 T > Cp.X143Gln), α2^cd59 (G➔A)^ (Hb Adana, c.179G > Ap.Gly60Asp), α^PA-2^ (c.*92A > G), and α2^cd142 (T➔A)^ (Hb Icaria, c.427 T > Ap.X143Lys) alleles were all found only among Kuwaitis. When the frequencies were computed based on the total number of families in the study, again, the PA-1 allele was most frequent at 0.0552, followed by the -α^-3.7 kb^ deletion at 0.18. This was approximately the same when only Kuwaiti families were considered.

## Discussion

The molecular diagnosis of α-thalassemia is not straightforward and requires resources that are not readily available in many centers. Moreover, it is economically unviable to provide this service to all suspected patients in many countries with a high prevalence. It is therefore necessary to have a good understanding of the prevalent genotypes and phenotypes, which will facilitate genetic counseling and the identification of patients who would best benefit from molecular diagnosis. This report is limited to patients who were suspected to have moderate to severe alpha thalassemia based on persistent microcytic and hypochromic anemia, no laboratory evidence of iron deficiency and normal hemoglobin electrophoresis or HPLC. It also included patients with H inclusion bodies and/or an H band on HPLC.

Given the selection criteria for screening patients in this study, it is not surprising that most patients had the HbH disease phenotype and that few had thalassemia trait or silent carrier status. A previous study from Kuwait showed that most alpha thalassemia silent carriers had Hb levels > 10 g/dl. Therefore, individuals with mild microcytosis and hypochromia, but with Hb > 10 g/dl and no HbH inclusions or bands, should not be considered for molecular diagnosis, but counseling should be offered after screening the family with CBC and HPLC to rule out β-thalassemia. Resources can then be channeled to individuals with a more severe outlook to identify those with HbH disease that need to be followed in the clinic.

This study revealed the extent of the diversity among individuals with alpha thalassemia in Kuwait. As expected, the vast majority of the patients had the α^PA-1^ nondeletional allele, which has been reported as the most common cause of HbH disease in Kuwait and in the region. The next most common allele was the –α^-3.7 kb^ deletion, although it was usually present as a compound heterozygote with other alleles. Interestingly, the third most frequent allele in this study was α^0^ (−-^MED^), which was found in 13 patients, in whom it mostly presented an HbH disease phenotype. The ^__MED^ deletion was in compound heterozygosity with α2^cd19-G^ in 6 patients from 2 related families, with –α^-3.7 kb^ in 2 patients and with α^IVS1-5nt^ and α^PA-1^ in 1 patient each.

The α^PA-1^ allele was first described in Eastern Saudi Arabia [10], and previous studies from Kuwait [2, 16] highlighted its role in the etiology of HbH disease among our patients. It has also been reported as the most common cause of HbH disease in Saudi Arabia [21], Jordan, [22], Bahrain [23] and the UAE [24]. The allele is therefore widespread in the Arabian Gulf, especially in the countries adjoining Saudi Arabia. It is uniformly associated with a moderate thalassemia intermediate phenotype with only occasional blood transfusion requirements, usually with intercurrent infection [16]. HbH disease is mainly a disease of childhood in Kuwait. Most patients are referred to the clinic for investigation of persistent microcytic and hypochromic anemia not responding to iron therapy. Indeed, the anemia tends to improve as the child stops rapidly growing and enters puberty.

This study is the first to report the presence of the α^0^ (−-^MED^) allele among Kuwaiti patients. It presented as compound heterozygotes with other α-thal alleles; however, the phenotype was mild with moderate anemia. The exceptions were 2 patients with the –^MED^/ α^cd19^α genotype, who presented with stunted growth and are on regular transfusion because of severe anemia. The other rare alleles found in the study included α^0^ (−-^FIL^) in a Filipino child, HbCS (α2^Cd 142 *TAA → CAA*^), Hb Adana (α^cd59G/A^), α^PA-2^ (*AATAAA→AATGAA*), and Hb Icaria (α^cd 142 *TAA → AAA*^).

## Conclusions

This study demonstrated the diversity of alpha thalassemia alleles among Kuwaitis. While the deletional allele is quite widespread, nondeletional α^PA-1^ is responsible for most moderate to severe HbH disease. Interestingly, the α^0^ (^__MED^) allele is also encountered, with implications for premarital genetic counseling to prevent the birth of babies with alpha thalassemia major (Bart’s hydrops fetalis).

## Data Availability

The dataset used and/or analyzed during the current study is available from the corresponding author on reasonable request.

## Abbreviations

PCR: Polymerase chain reaction
PA: Polyadenylation
MED: Mediterranean
FIL: Filipino
SEA: Southeast Asia
Kb: Kilobases
Hb: Hemoglobin
CS: Constant Spring
CBC: Complete blood count
EDTA: Ethylenediaminetetraacetic acid
SD: Standard deviation
ANOVA: Analysis of variance
UAE: United Arab Emirates
